# Developing a procedure mimicking transvaginal mesh implantation in women in a modified POP rat model

**DOI:** 10.3389/fmed.2025.1603161

**Published:** 2025-07-30

**Authors:** Lulu Wang, Fang Long, Keqing Yan, Lutong Li, Na Gao, Deqiong, Zhen Xiao

**Affiliations:** ^1^Department of Obstetrics and Gynecology, First Affiliated Hospital of Dalian Medical University, Dalian, China; ^2^People's Hospital of Nagqu, Nagqu, Tibet, China; First Affiliated Hospital of Dalian Medical University, Dalian, Liaoning, China; ^3^Department of Obstetrics and Gynecology, Zhuzhou People's Hospital, Zhuzhou, Hunan, China; ^4^Department of Obstetrics and Gynecology, Maternal and Infant Hospital, Yingkou Economic and Technological Development Zone, Yingkou, Liaoning, China; ^5^Women and Children's Hospital of Tibet Autonomous Region, Tibet, China

**Keywords:** pelvic organ prolapse, animal model, biomechanical, transvaginal mesh, rat

## Abstract

**Introduction:**

This study aims to establish a simple and reproducible transvaginal mesh surgery rat model based on the modified pelvic organ prolapse rat model.

**Methods:**

A total of 24 10-week-old female nulliparous Wistar rats were used in this study. The control group consisted of six rats with no interventions. The ovariectomy group included six rats that underwent bilateral ovariectomy. The pelvic organ prolapse group comprised 12 rats that underwent cervical pendant modeling 2 weeks after bilateral ovariectomy. Fourteen days post-modeling, six rats from the pelvic organ prolapse group underwent transvaginal mesh surgery. The rat pelvic organ prolapse quantification system was used to evaluate the prolapse condition of the rats before and after pelvic organ prolapse modeling, as well as after transvaginal mesh surgery. Vaginal wall tissue was collected to assess biomechanical changes before and after pelvic organ prolapse modeling. Additionally, vaginal wall and sacral ligament tissues were collected to evaluate structural changes and collagen alterations before and after pelvic organ prolapse modeling.

**Results:**

The pelvic organ prolapse rat model exhibits anatomical prolapse, biomechanical changes, and pathological changes, including collagen fiber rupture and reduced collagen density. In contrast, the transvaginal mesh rat model demonstrates anatomical recovery in prolapsed rats.

**Conclusion:**

This study successfully modified the pre-existing rat model of pelvic organ prolapse and effectively mimicked human transvaginal mesh surgery using this model.

## Brief summary

A POP rat model was modified based on our previous one. Additionally, the new model was used to mimic transvaginal mesh surgery.

## Introduction

Animal models are extensively employed in the study of female pelvic organ prolapse (POP) to investigate pathogenesis, exploring surgical interventions, and testing new materials. Despite the unclear pathogenesis of POP, it is evident that the condition follows a chronic, progressive course. Utilizing animal models addresses the complexity and ethical concerns related to studying this disease effectively. The primary animal models for POP include mice (1–3), rats (4, 5), rabbits (6, 7), sheep (8–10), rhesus macaques (11, 12), and squirrel monkeys (13). Although rodents are small, their daily activities are mainly prostrate, and their morphology and pelvic floor structure stress are different from humans; they are widely used because of their short estrous cycle, similar anatomical structure to humans, and low cost (14).

Based on our previously developed POP rat model (15), we have created a refined version that addresses several limitations of the original model. Previously, the pelvic organ prolapse model was mainly established by cervical negative pressure maintenance. In this study, we used a cervical gravity suspension device. The enhancements are as follows: first, during frequent negative pressure suction, the suction device may slip and cause vaginal wall and even urethral injury. This study used a new cervical exposure device as well as a cervical fixation device. There was little damage to the tissues around the cervix during the operation. Second, artificial traction differs significantly from the vertical gravitational forces experienced by patients with pelvic organ prolapse. In this study, rats were subjected to the same vertical gravity through the gravity of the cervical suspension device. Finally, it was challenging to accurately model the condition in the majority of affected individuals, especially the postmenopausal population. In this study, the POP rat model was added with bilateral ovariectomy to mimic the menopausal state. Therefore, considering these aspects, we optimized and developed a less invasive and more natural model.

To the best of our knowledge, transvaginal mesh (TVM) surgery has yet to be simulated in rodent models, significantly impeding *in situ* biomaterial testing. As a minimally invasive procedure, TVM has demonstrated significant efficacy in treating POP; however, due to complications associated with mesh exposure, its widespread application is limited (16). The underlying mechanism of mesh exposure remains unclear; hence, preclinical experiments for evaluating implant materials are indispensable. Currently, rodent studies primarily involve placing mesh in the abdominal or vaginal walls (1, 3, 17), which fails to accurately simulate the human TVM procedure. Consequently, we aim to establish a rat model of TVM and study the mechanism of mesh implantation-related complications, and ultimately to develop more effective and less complicated treatments for pelvic organ prolapse.

## Materials and methods

### Animals

**Figure 1:**
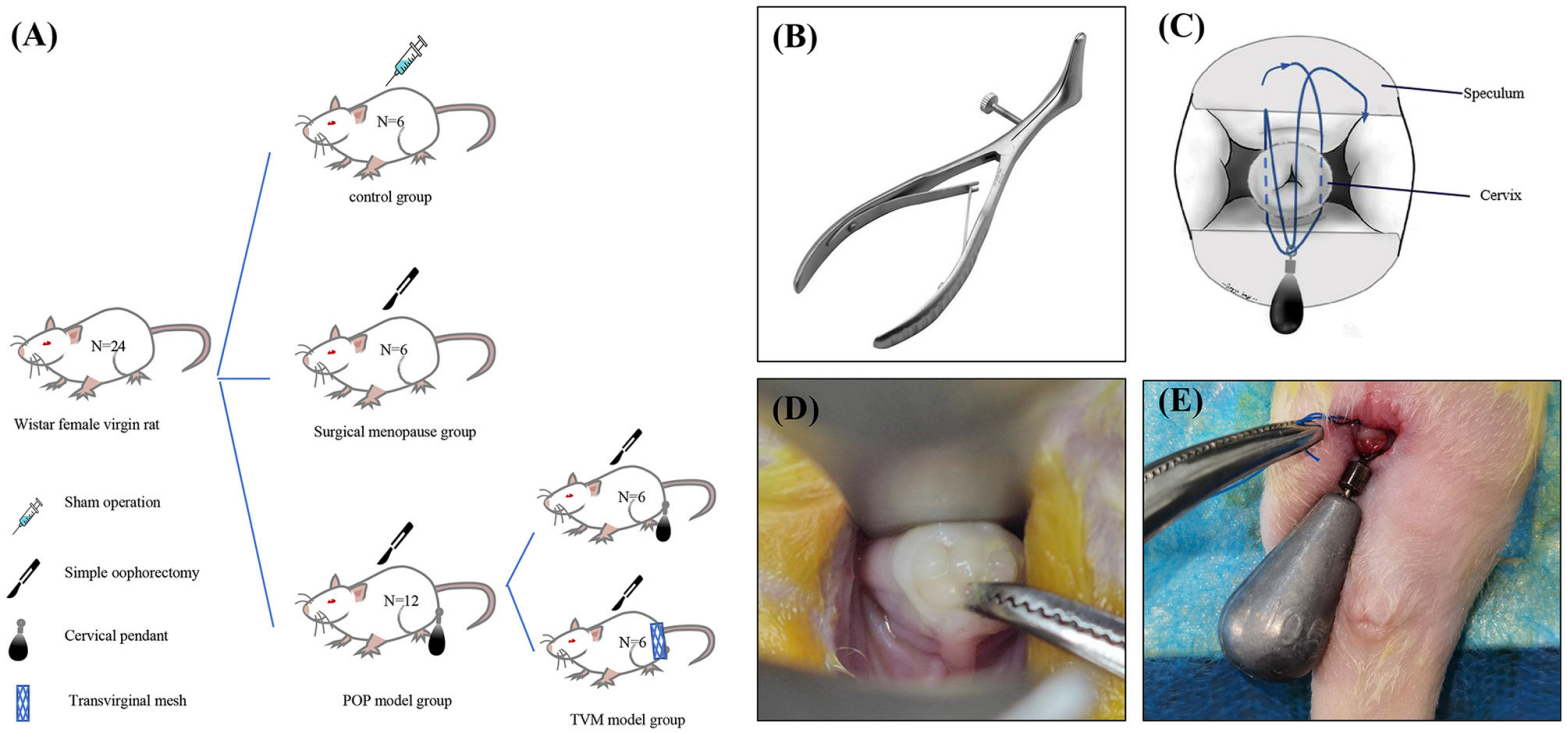
Schematic diagram illustrating the establishment of a rat model for POP and specific methodologies. **(A)** Experimental grouping; **(B)** modified vaginal speculum for rats; **(C)** schematic representation of the modeling technique; **(D)** rat cervix exposed under the speculum; **(E)** schematic depiction of the post-modeling cervix.

This study adhered to laboratory animal welfare regulations and was approved by the ethics committee on Laboratory Animals of Dalian Medical University (No. AEE24065). Ten-week-old nulliparous female Wistar rats were obtained (*n* = 24), with a mean weight of 207.88 ± 19.77 g. The rats were housed in an animal facility with standard humidity (45%−50%) and temperature (22 ± 2°C) under a 12-h light/dark cycle. All animals had access to standard food and water and were fasted for 12 h before the study procedure, with free access to water. The entire experiment was conducted under sterile conditions. The 24 Wistar rats were randomly assigned to three groups: the control group rats were sacrificed and tested without any intervention (*n* = 6); the ovariectomy (OVX) rats were sacrificed 4 weeks after bilateral ovariectomy (*n* = 6); 2 weeks after bilateral ovariectomy, half of the POP group rats were sacrificed after successful modeling (*n* = 6), the remaining half of POP rats underwent TVM (*n* = 6) (Figure 1A).

### POP rat model modification

Our previously developed rat POP model has been modified to reduce invasiveness and better mimic the human menopausal state of pelvic organ prolapse. First, we introduced a method for cervical exposure using a novel vaginal speculum that closely resembles its human counterpart to expose the rat cervix (Figure 1B). This technique facilitates the manipulation of the cervix and vagina, including cervical traction and the separation of vaginal wall tissues from para-vaginal tissues, while ensuring sufficient cervical visibility. Second, all POP rats underwent bilateral ovariectomy before the modeling process to replicate the hormonal changes associated with menopause. Finally, after the modeling procedure, all POP rats were housed in elevated cages (length: 380 mm, width: 280 mm, height: 310 mm) to ensure that the gravitational force exerted by the cervical weight device was aligned parallel to the body axis, thus more accurately simulating human pelvic organ prolapse.

### Approaches to the modeling of pelvic organ prolapse

The rats underwent a 12-h fasting period before surgery, during which water was not restricted. Anesthesia was induced via intraperitoneal injection of pentobarbital sodium. Once a deep anesthetic state was achieved, the rats were positioned supine on the operating table, and the surgical site was sterilized and prepared. A vaginal speculum was used to visualize the cervix, and any cases of abnormal development, including cervical deformities or agenesis that might compromise the experimental results, were excluded. The cervix was gently pulled to the level of the vaginal aperture using right-angle forceps (Figure 1D). A 10.5 g tungsten steel weight was securely attached at the level of the cervical aperture with a 3-0 non-absorbable suture, and the vagina, perineum, and perianal regions were re-sterilized (Figures 1C–E). Subsequently, the rats were transferred to transport cages for anesthesia recovery. On the day after surgery, they were provided with a residue-free diet, and from the first postoperative day onward, they were housed in tall medium cages with free access to standard food and water.

### The TVM surgery simulating in the POP rat model

Three materials were used for prolapse implantation: polypropylene mesh (PP, *n* = 2), polycaprolactone scaffolds (PCL, *n* = 2), and decellularized porcine small intestinal submucosa scaffolds (SIS, *n* = 2). Each mesh measured 6 cm × 0.3 cm. Before use, the mesh underwent a comprehensive sterilization protocol: a 30-min soak in 75% ethanol, followed by three 1-min rinses with phosphate-buffered saline, and finally, a 30-min exposure to ultraviolet (UV) light.

**Figure 2:**
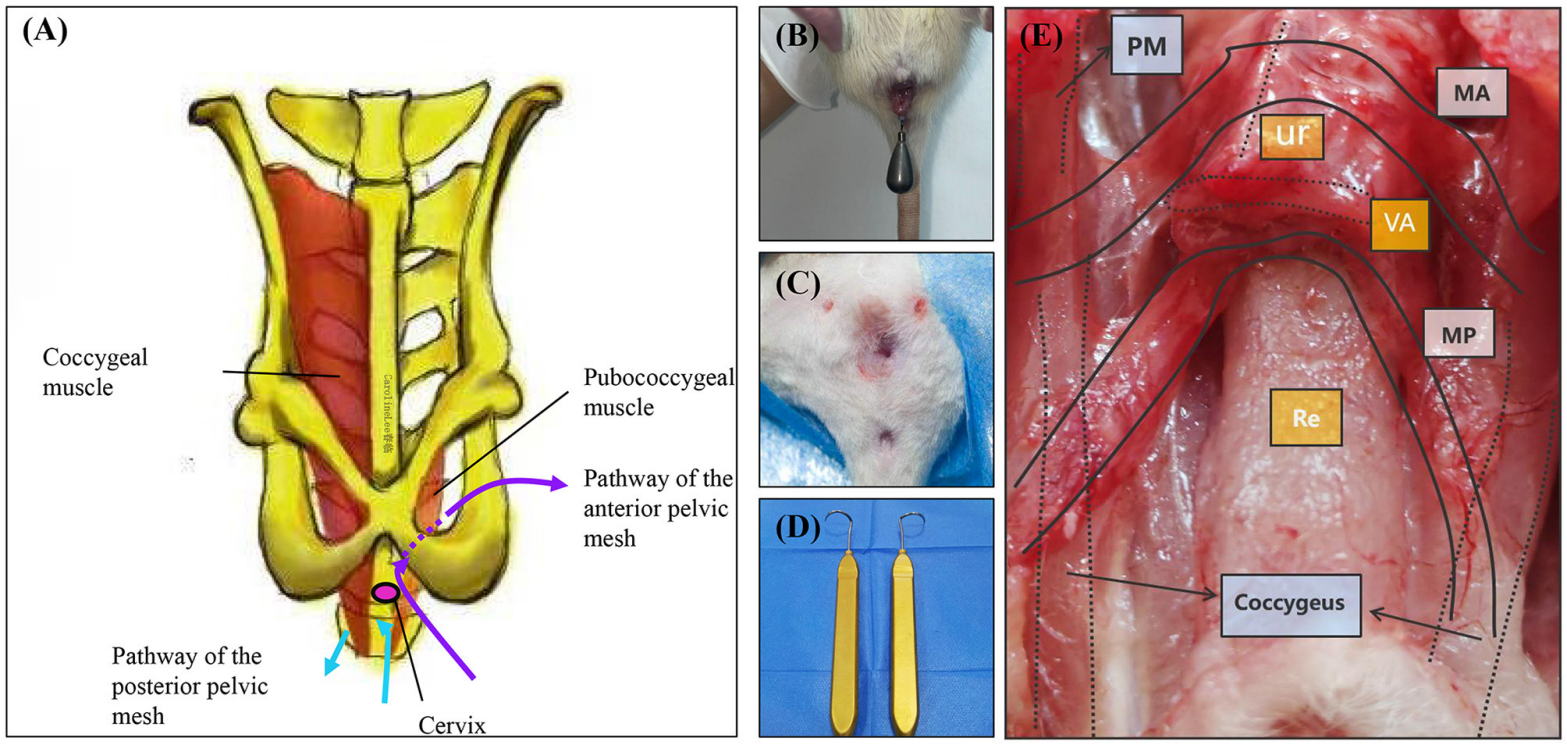
TVM protocol and comparison map. **(A)** Schematic diagram illustrating the anatomical structure of rat TVM and mesh alignment. **(B)** Preoperative perineal condition of rats undergoing TVM. **(C)** Postoperative perineum after TVM in rats. **(D)** Puncture guide needle. **(E)** Gross anatomical and mesh trajectory diagram following TVM surgery in rats. PM, Pubococcygeus muscle; MA, Anterior pelvic mesh trajectory path; MP, Posterior pelvic mesh trajectory path; Ur, Urethra; VA, Vagina; Re, Rectum.

The rats underwent a 12-h fasting period before surgery, during which water was not restricted. Anesthesia was induced via intraperitoneal injection of pentobarbital sodium. Once a deep anesthetic state was achieved, the rats were positioned supine on the operating table, and the surgical site was sterilized and prepared. Two sets of skin markers were created: the first set was positioned 0.3 cm below the midpoint of the bilateral superior pubic rami, and the second set was placed 0.3 cm below the midpoint of the bilateral coccygeal muscles. A longitudinal incision of approximately 0.5 cm was made at the midpoint of the anterior fornix upward and the midpoint of the posterior fornix downward. Subsequently, the incision was bluntly separated bilaterally: the anterior vaginal wall was separated medial to the descending pubic ramus on both sides, and the posterior vaginal wall was separated lateral to the ischiococcygeal muscles on both sides. The puncture guide needle was used to penetrate the skin from the incision of the anterior vaginal wall along the right tissue space from the right side of the first set of marks. The implant material fixed with 6-0 silk thread was attached to the tip of the puncture guide needle, and the needle was withdrawn to remove the implant material. The same procedure was repeated on the left side. The puncture guide needle was then used to penetrate the skin through the incision of the posterior vaginal wall along the right tissue space from the right side of the second set of marks. The implant material was again fixed with 6-0 silk thread, attached to the needle tip, and the needle was withdrawn to remove the implant material. The left side underwent an identical procedure. The position of the implant material was adjusted to ensure it was free of tension, followed by suturing of the anterior and posterior vaginal walls using 7-0 absorbable thread. The rats were transferred to transport cages for anesthesia recovery, provided with a residue-free diet on the day after surgery, and given standard food and water from the first postoperative day onwards (Figure 2).

### Sample collection

Tissue samples from the vaginal wall and sacral ligaments were collected at the start of the experiment, 2 weeks after bilateral oophorectomy, and 2 weeks after pelvic organ prolapse modeling (excluding those treated with TVM). All specimens were stored at −80°C for later histological staining and biomechanical testing.

### Rat pelvic organ prolapse quantification system

An accurate and reproducible quantitative system is essential for the objective evaluation of the progression of pelvic organ prolapse in animal models. We have implemented the previous Rat Pelvic Organ Prolapse Quantification (ROPQ) system (15) and concurrently enhanced the relevant regulations. The degree of pelvic organ prolapse was quantified for each classification (Table 1). All rats in the POP group were assessed utilizing the ROPQ system.

**Table 1:** The rat pelvic organ prolapse quantification system.

**Parameter**	**Measurement**
Degree of perineal bulge	0 = None
	1 = Slight bulge, visible vagina but no cervix
	2 = Moderate size bulge, visible vagina and partial cervix, but cervix do not coming out
	3 = Severe bulge, cervix coming out
	4 = Vagina coming out
Anal prolapse	0 = None
	1 = Slight bulge, visible dentate line
	2 = Moderate size bulge, visible rectal mucosa
	3 = Severe bulge, cervix coming out
Distance from cervix to vaginal introitus^a^	mm
Length of the perineal body^b^	Mm
Genital hiatus^c^	mm

^a^Distance between cervix and vaginal introitus was measured as the distance from the plane of the cervix to the plane of the vaginal outlet in millimeters; ^b^The perineal body was measured in millimeters from the posterior fourchette to the midanus; ^c^Genital hiatus: The genital hiatus was measured in millimeters from the anterior to the posterior vaginal walls at the level of the introitus.

### Biomechanical testing

At the end of the experiment, three rats from the control group, three rats from the OVX group, and three rats from the POP group were collected for biomechanical testing of vaginal wall tissue. The vaginal wall tissues from each group were tested using a uniaxial testing machine. Before each test, the samples were preloaded to 0.05 N, followed by 10 cycles of preconditioning at an elongation rate of 25 mm/min within an elongation range of 0 to 2 mm, corresponding to the elastic region of the load-elongation curve. Immediately following the preconditioning, uniaxial tensile tests to failure were conducted at the same elongation rate. Data points were collected at intervals of 0.01 s, and the failure modes were recorded using the provided software and subsequently imported into Excel for further analysis. The ultimate load (N) at failure was defined as the maximum load sustained by the target tissue before failure.

### Histological staining

The specimens were fixed in 4% paraformaldehyde at ambient temperature (25°C) and subsequently embedded in paraffin. All samples were sectioned to a thickness of 5 micrometers. Hematoxylin and eosin (H&E) staining was conducted to examine alterations in tissue morphology. Masson's trichrome staining was employed to assess tissue morphology and total collagen expression. Sirius red staining was utilized to determine the content of immature and mature collagen fibrils. Under polarized light microscopy, stained collagen fibers exhibited various birefringence patterns, with type III collagen fibrils appearing green and type I collagen fibrils ranging from yellow to orange/red. Quantitative analysis of the images was performed using ImageJ software.

### Statistical analysis

Statistical analyses and graphical representations were performed using GraphPad Prism 10.3.0 (GraphPad Software Inc., USA). Data are presented as mean ± standard deviation. A two-tailed Student's *t*-test was used to assess differences between the two groups. Repeated measures analysis of variance was used to evaluate differences in a variable over time. Statistical significance was defined as *P* < 0.05.

## Results

### The postoperative physiological state of rats

**Figure 3:**
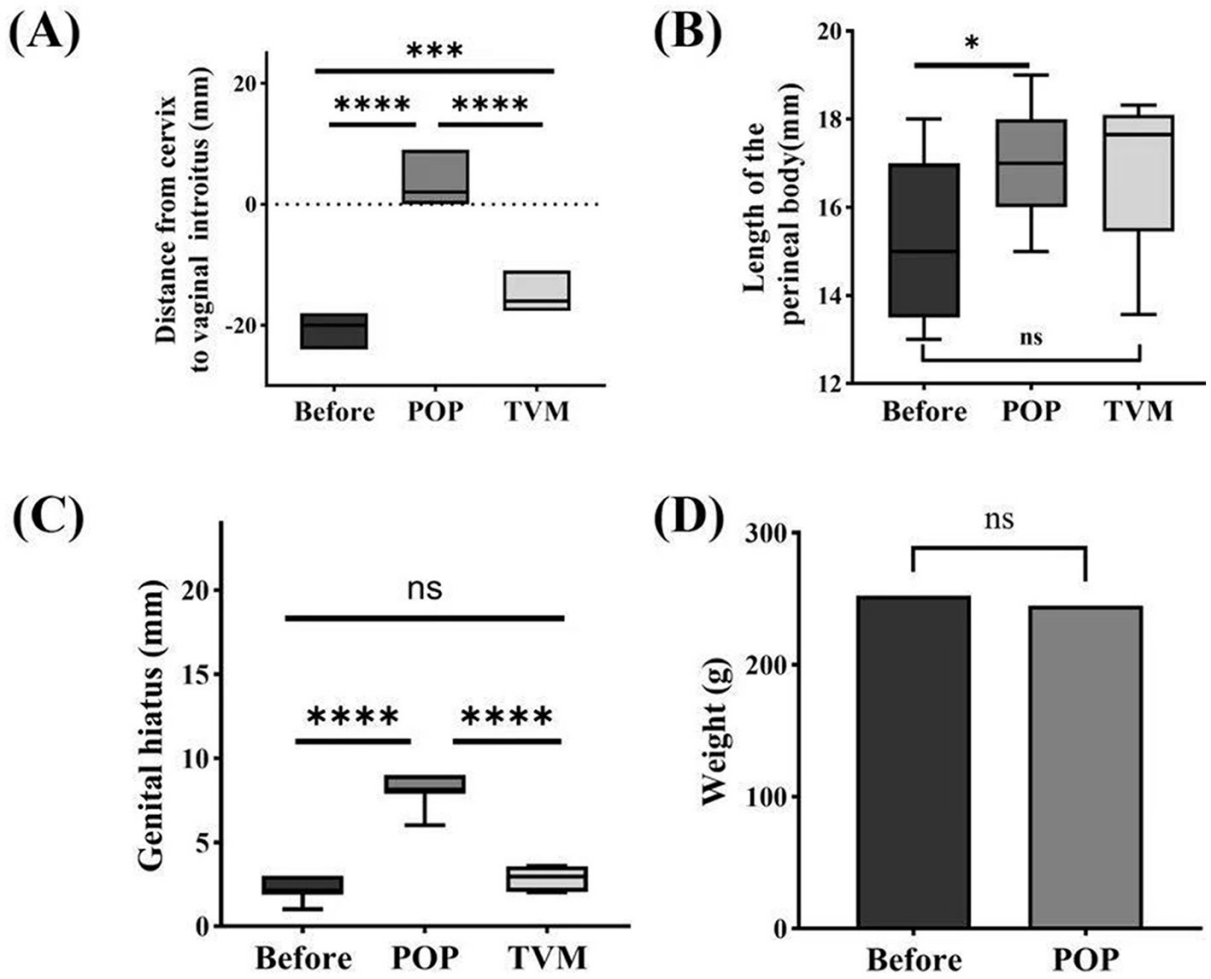
Systematic evaluation of ROPQ in rats: **(A)** the distance between the cervix and vaginal orifice; **(B)** perineal body length; **(C)** reproductive hiatus size; **(D)** changes in weight before and after Pelvic Organ Prolapse Modeling. Significant differences are denoted by ^*^P < 0.05; ^***^P < 0.001; ^****^P < 0.0001.

Before modeling, the mean weight of the POP group rats was 252.33 ± 23.05 g, whereas post-modeling, their mean weight was 244.92 ± 23.38 g. Comparative analysis revealed no statistically significant difference in weight between the pre-modeling and post-modeling stages (*P* = 0.4422) (Figure 3D). The cervical gravity device fell off in two rats in the POP group on the 5th and 6th postoperative days, respectively, with no recurrence observed following re-operation. Ten rats exhibited increased vaginal discharge post-surgery, and two of these developed vulvar ulcers; however, all recovered following disinfection and antimicrobial therapy. One rat that was implanted with PCL died 13 weeks post-TVM.

### ROPQ system

The ROPQ system demonstrated that the prolapse in rats progressively aggravated following POP modeling compared to preoperative conditions, with a perineal prolapse grade of 3 or higher observed on the 14th day. The distance from the cervix to the vaginal orifice was−19.75 ± 1.60 mm before POP modeling and 2.83 ± 2.86 mm following POP modeling (*P* < 0.0001). After TVM, this distance was measured at −15.33 ± 2.70 mm, with statistically significant differences compared to both pre-POP and post-POP conditions (*P* < 0.0001) (Figure 3A). The perineal body measured 15.42 ± 1.83 mm before POP modeling and increased to 17.00 ± 1.28 mm post-POP modeling (*P* = 0.0225). Following TVM, the perineal body measurement was 16.95 ± 1.92 mm, showing no statistically significant difference compared to the pre-POP modeling measurement (*P* = 0.1416) or the POP group (*P* = 0.9502) (Figure 3B). The genital hiatus measured 2.25 ± 0.62 mm before POP modeling and increased to 8.17 ± 0.83 mm post-POP modeling (*P* < 0.0001). Following TVM, the genital hiatus was recorded at 2.83 ± 0.76 mm, significantly different from both pre-POP and post-POP measurements (*P* < 0.0001) (Figure 3C).

### Biomechanical testing

**Figure 4:**
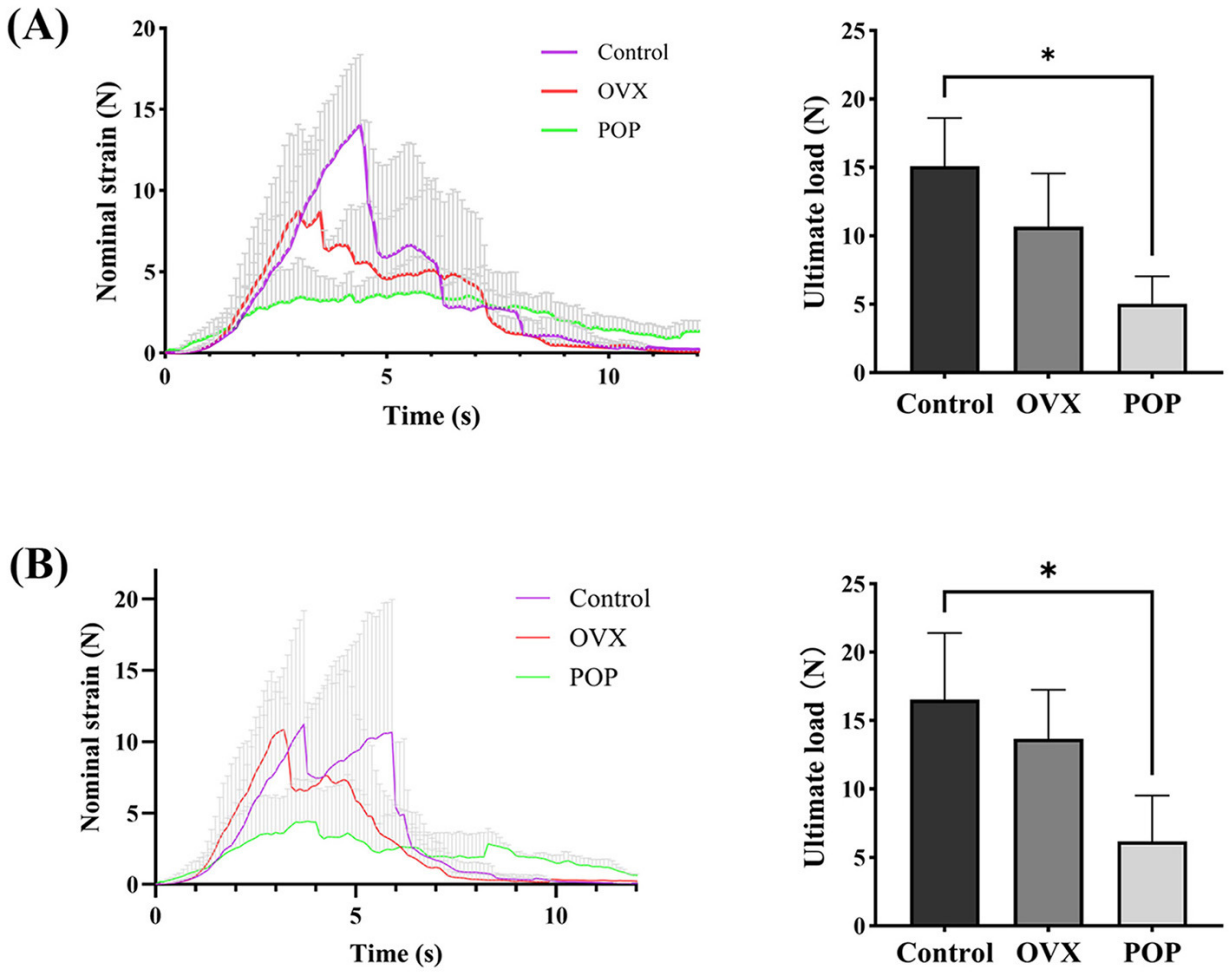
Biomechanical testing of the vaginal wall: **(A)** strain and ultimate load of the anterior vaginal wall tissue; **(B)** strain and ultimate load of the posterior vaginal. Significant differences are denoted by ^*^P < 0.05.

The results of the mechanical testing indicated that the ultimate load of the anterior vaginal wall was 15.10 ± 3.50 N in the control group and 10.67 ± 3.90 N in the OVX group (*P* = 0.2169). In the pelvic organ prolapse (POP) group, the ultimate load of the anterior vaginal wall was 5.03 ± 2.02 N, which was significantly lower than that in the control group (*P* = 0.0125). No significant difference was observed between the OVX and control groups (*P* = 0.0901) (Figure 4A). For the posterior vaginal wall, the ultimate load was 16.54 ± 4.88 N in the control group and 13.67 ± 3.59 N in the OVX group (*P* = 0.4582). In the POP group, the ultimate load of the posterior vaginal wall was 6.17 ± 3.35 N, which was also significantly lower compared to the control group (*P* = 0.0386). However, no significant difference was found between the OVX and control groups (*P* = 0.0572) (Figure 4B).

### Histopathology evaluation

H&E staining indicated a notably loose tissue structure in the sacral ligament and anterior vaginal wall of the POP group. Furthermore, Masson's trichrome staining highlighted a markedly disorganized arrangement of collagen fibers in the POP group.

**Figure 5:**
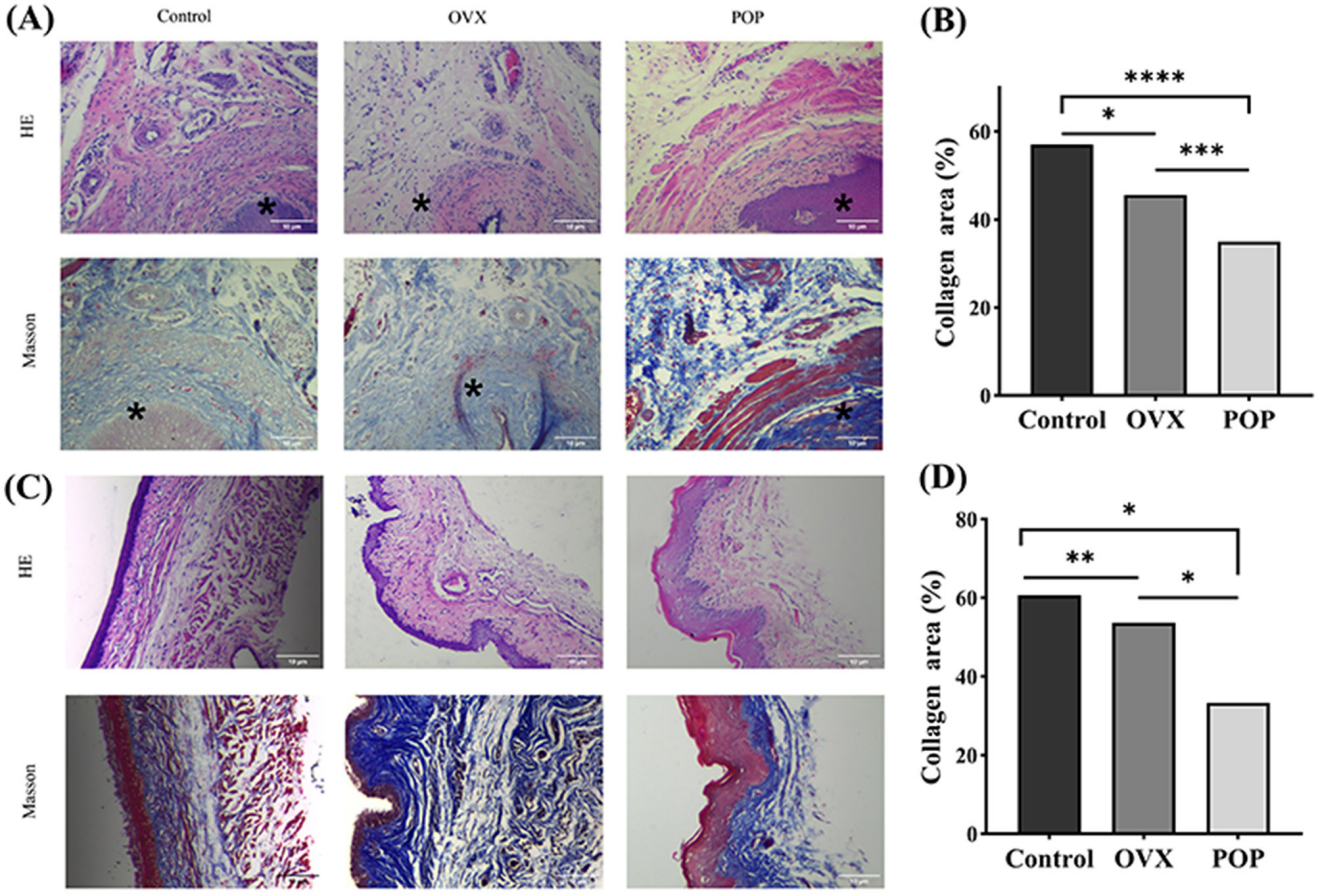
The sacral ligament and vaginal wall tissue were subjected to 40 × microscopic HE staining and Masson staining. *, Cervix. **(A)** HE staining and Masson staining of the sacral ligament in the control group, OVX group, and POP group; **(B)** HE staining and HE staining of the sacral ligament in the control group, OVX group, and POP group; **(C)** statistical chart depicting the proportion of collagen in the sacral ligament; **(D)** Statistical plot illustrating the proportion of collagen in the anterior vaginal wall. Significant differences are denoted by ^*^P < 0.05; ^**^P < 0.01; ^***^P < 0.001; ^****^P < 0.0001.

Masson's trichrome staining quantification results indicated that for the sacral ligament tissue, the collagen content in the control group was 57.03 ± 6.04%, while in the OVX group, it was 45.63 ± 3.33%, with a statistically significant difference (*P* = 0.0163). The collagen content in the POP group was 35.05 ± 2.52%, which was significantly lower than the control group (*P* < 0.0001) and the OVX group (*P* = 0.0004). For the anterior vaginal wall, the collagen content was 60.44 ± 1.23% in the control group, 53.38 ± 2.17% in the OVX group (*P* = 0.008), and 33.21 ± 11.84% in the POP group (*P* = 0.0167), showing a significant reduction compared to the control group. The difference between the POP group and OVX group was also statistically significant (*P* = 0.0441) (Figure 5).

**Figure 6:**
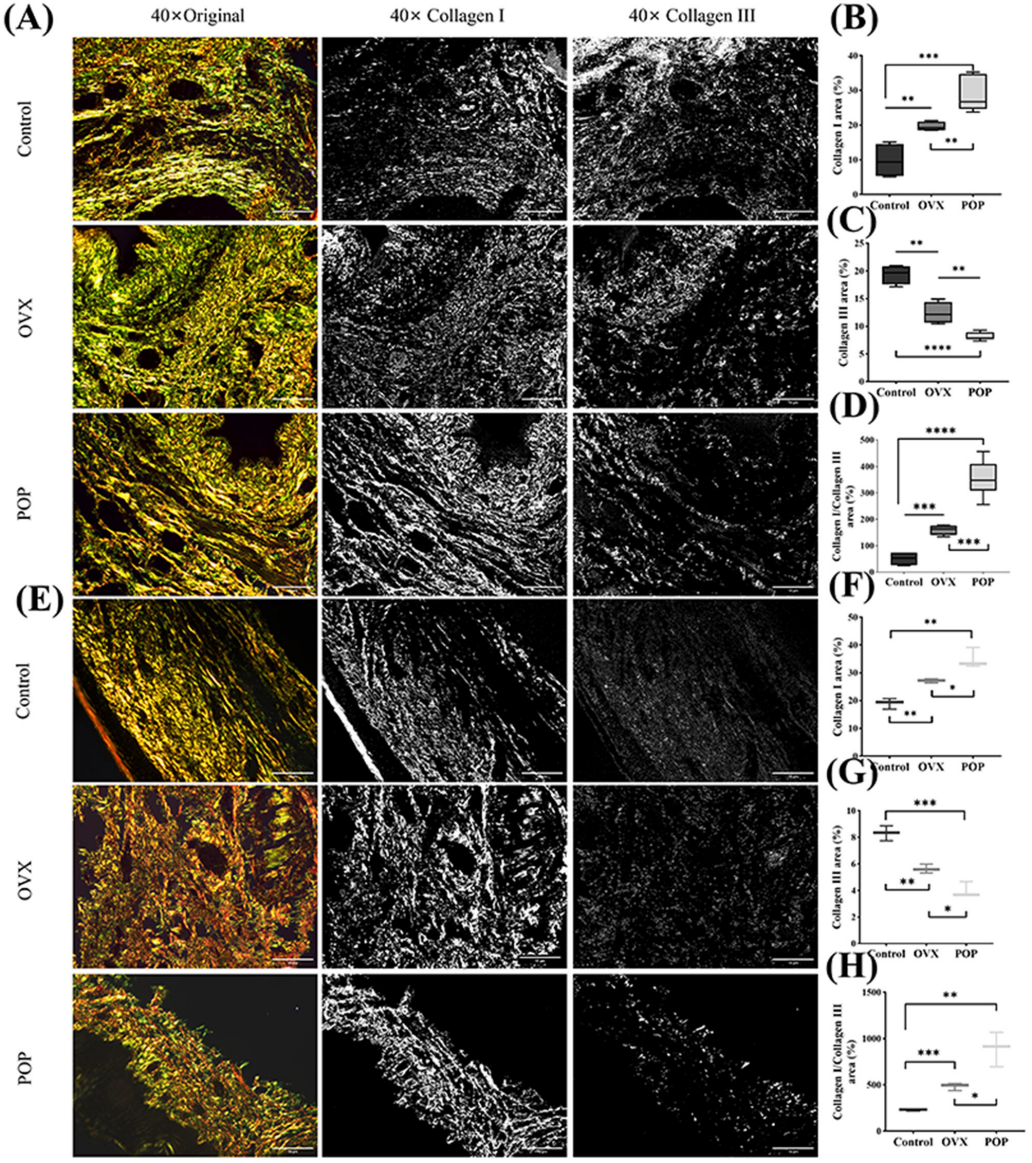
The collagen composition and arrangement in the sacral ligament and anterior vaginal wall were examined using polarized light microscopy after staining with Sirius red. **(A)** The Sirius red staining of the sacral ligament tissue; **(B)** collagen I in the POP group was significantly higher than that in the control and OVX groups; **(C)** collagen III in the POP group was significantly lower than that in the control and OVX groups; **(D)** the ratio of Col I/Col III in the POP group was significantly higher than that in the control and OVX groups; **(E)** the Sirius red staining of the anterior vaginal wall tissue; **(F–H)** the results of Col I, Col III, and Col I/Col in anterior vaginal wall were similar with the sacral ligament tissue. Significant differences are denoted by ^*^P < 0.05; ^**^P < 0.01; ^***^P < 0.001; ^****^P < 0.0001.

Under polarized light microscopy, type I collagen appeared yellow or bright orange, while type III collagen appeared green. The content of type I collagen in the control group was 9.75 ± 4.87%, and in the OVX group, it was 19.6 ± 1.32% (*P* = 0.0079). In the POP group, it was 28.69 ± 4.99%, which was statistically significant compared to the control group (*P* = 0.0003) and the OVX group (*P* = 0.0081). The quantitative results of type III collagen in the sacral ligament showed that the control group had 19.34 ± 1.73%, and the OVX group had 12.41 ± 1.94%, with a statistically significant difference between the two groups (*P* = 0.0018). The POP group had 8.14 ± 0.74%, which was statistically significant compared to the control group (*P* < 0.0001) and the OVX group (*P* = 0.0011). Subsequently, the ratio of type I collagen to type III collagen was calculated, showing that the control group had 204.48 ± 26.15%, the OVX group had 639.96 ± 18.75%, with a statistically significant difference between the two (*P* = 0.0005), and the POP group had 2127.66 ± 67.20%, which was significantly different compared to the control group (*P* < 0.0001) and the OVX group (*P* = 0.0005). Similarly, the anterior vaginal wall tissue was quantified. The content of type I collagen in the anterior vaginal wall was 19.02 ± 1.94% in the control group, 27.11 ± 0.71% in the OVX group (*P* = 0.0025), and 34.94 ± 3.67% in the POP group (*P* = 0.0027), which was significantly higher than that in the control group. The difference between the POP and OVX groups was also statistically significant (*P* = 0.0222). In the anterior vaginal wall, the content of type III collagen was 19.34 ± 1.73% in the control group, 12.41 ± 1.94% in the OVX group (*P* = 0.0021), and 8.14 ± 0.74% in the POP group (*P* = 0.0008), which was significantly lower than that in the control group. The difference between the POP and OVX groups was also statistically significant (*P* = 0.0141). Subsequently, the ratio of type I collagen to type III collagen was found to be 19.34 ± 1.73% in the control group, 12.41 ± 1.94% in the OVX group (*P* = 0.0004), and 8.14 ± 0.74% in the POP group (*P* = 0.0036), which was statistically significant compared to the control group and the OVX group (*P* = 0.0208) (Figure 6).

## Discussion

The establishment of animal models for the diagnosis and treatment of POP diseases is highly indispensable. First, the pathogenesis of POP remains unclear, and human experiments and tissue acquisition are subject to ethical constraints. Hence, it is essential to develop appropriate animal models to investigate the pathogenesis of POP diseases and obtain targeted treatment approaches. Second, the prevailing main treatment modality for POP is mesh surgery and traditional autologous tissue implantation surgery (18, 19). The implanted mesh gives rise to numerous complications, mainly encompassing mesh exposure and pain (16, 20), which is the key factor limiting its application. Nevertheless, the anatomical cure rate of mesh surgery is significantly higher than that of traditional autologous tissue implantation surgery. It is extremely necessary to study the pathogenesis of mesh complications to reduce their incidence in the current treatment of POP. Similarly, the development of animal models will bring greater convenience. This study commences from the above two aspects to explore new rat models of POP and TVM models.

This study successfully established a rat model of pelvic organ prolapse (POP) and comprehensively evaluated its feasibility through anatomical, pathophysiological, and histological assessments. Specifically, the ROPQ system evaluation revealed that by day 14 post-operation, POP rats exhibited grade three to four perineal prolapse and grade two anal prolapse, indicating a significant three-level anatomical prolapse effect. Histological analysis via HE and Masson staining demonstrated that compared to the control and OVX groups, the sacral ligament and anterior vaginal wall tissues in the POP group were notably looser. Biomechanical studies further confirmed that the anti-tensile strength of vaginal wall tissue in the POP group was significantly weaker than in the control group, consistent with the anatomical, physiological, and pathological changes observed in human POP. Additionally, monitoring post-modeling physiological conditions showed no severe complications in the rats, confirming the safety of this method. Various experimental animals are used in POP research, including rodents, rabbits, sheep, and non-human primates. Non-human primates are considered ideal due to their similarity to humans in the delivery process, reproductive cycles, and pelvic floor anatomy (11). However, ethical concerns and high experimental costs limit their use. Rodents, with short reproductive cycles and low breeding requirements, are currently the most widely used. Nonetheless, specific methods vary, such as vaginal dilation, ovariectomy, nerve injury, and gene knockout, each with inherent limitations. For instance, vaginal dilation may cause acute damage.

The POP model in this study represents an innovative and enhanced version of previous models, aiming to improve both efficacy and convenience. First, an improved rat vaginal speculum was utilized to expose the cervix. All cervical procedures were conducted under direct visualization provided by the speculum, mirroring the exposure method used in human gynecological examinations. This approach minimizes damage to the vaginal wall and surrounding organs and reduces potential interference with subsequent research outcomes. Second, before modeling, all rats in the POP group underwent bilateral ovariectomy to simulate the postmenopausal state, reflecting the fact that pelvic organ prolapse predominantly occurs in postmenopausal women. Third, one potential cause of POP is chronic increased abdominal pressure. In this study, a gravity device was attached to the cervix to induce a sustained pulling force. To simulate the upright posture of humans, all rats in the POP group were housed in elevated cages, ensuring that the force direction on the cervix was perpendicular to the ground. Additionally, an appropriate level of force was applied to better mimic chronic pressure. Through preliminary experiments, it was determined that a 10.5 g gravity device could produce a significant effect while avoiding irreversible acute injury to rat tissues. Moreover, given that the cervical tissue of rats is significantly smaller than the gravity device, leading to a higher drop-off rate, we continuously optimized the fixation method, thereby reducing the likelihood of detachment and ensuring a continuous and stable gravitational effect.

The distribution of collagen in the sacral ligaments and anterior vaginal wall tissues of POP rats exhibits a distinct trend. Through Masson and Sirius Red staining of the sacral ligament and vaginal wall tissues from all rats, we observed that the severity of prolapse in POP rats was significantly greater than in the OVX group. As prolapse worsened, the total collagen content in both the sacral ligaments and vaginal wall tissues progressively decreased, while type I collagen increased and type III collagen decreased. Type I and type III collagens are primary components of the extracellular matrix, with type III collagen being particularly predominant in rat vaginal wall tissues, which is consistent with the results reported by Moalli et al. (21). In our study, the ultimate load of the vaginal wall tissue in the POP group was significantly lower than that in the control group, indicating that the biomechanical properties of the vaginal wall in the POP group were decreased. We hypothesized that increased synthesis of type I collagen, which has the function of providing tensile strength and stiffness, may be a compensatory mechanism to counteract the decline in the mechanical properties of the vaginal wall. However, the distribution of collagen in the pelvic floor tissue of POP patients is opposite. We hypothesize that the time of POP in humans is very long, and the compensatory increase of type I collagen may occur in the early stage of the disease, but after a long time of disease progression, the compensatory increase of type I collagen reaches the limit or even decompensates, so that the expression of type I collagen in the pelvic floor tissue of POP women is reduced.

The TVM rat animal model developed in this study demonstrates significant efficacy in the anatomical recovery of POP rats. Given the structural differences between the rat and human pelvis, notably the absence of an ischial structure in rats, we established two distinct pathways for mesh implantation: through the anterior vaginal wall and through the posterior vaginal wall. The mesh implanted via the anterior vaginal wall traverses the anterior vaginal space and exits through the obturator foramen along the pelvic fascia tendinous arch, mimicking human anatomy; conversely, the mesh implanted via the posterior vaginal wall passes behind the coccygeus muscle through the posterior vaginal space, which diverges from human anatomy. Utilizing the ROPQ system to evaluate the rats before POP modeling, post-POP modeling, and post-TVM modeling, we observed that rats exhibited significant perineal and anal prolapse following POP modeling. However, after TVM repair, perineal prolapse was markedly alleviated, thereby strongly validating the effectiveness of the TVM rat model in anatomical treatment.

The primary limitation of the POP model in this study is that the POP rats did not develop more severe anal prolapse. This may be attributed to the cervical sling maintaining the cervix in a vertical position relative to the ground. However, during locomotion, the posterior vaginal wall and anus are pulled toward the anterior vaginal wall by the cervix, thereby mitigating the severity of anal prolapse. In addition, the modeling period of POP is short, which may be different from the long prolapse formation in humans. It must be acknowledged that one of the limitations of the TVM model is its focus on vaginal mesh. In many countries, vaginal mesh is rarely used because of the risk of mesh exposure and other mesh-related complications. This limitation of clinical utility means that the relevance of our findings to regions where mesh is not common may be limited. In addition, the controversy surrounding mesh use highlights the need for continued research into alternative therapies and improved mesh techniques to address these complications. Our study highlights the importance of understanding the mechanisms of mesh-related complications to develop safer and more effective surgical interventions. Given the strengths and limitations of this study, it may be appropriate to apply the POP and TVM models in specific types of research. For instance, the biocompatibility assessment of exogenous implants, such as synthetic meshes and biological meshes, could potentially adopt these models. These models are also applicable for evaluating the efficacy of stem cell transplantation or immunotherapy. To the best of our knowledge, this is the first study to simulate human transvaginal whole pelvic reconstruction in rats. Nevertheless, it should be emphasized that this study represents only a preliminary investigation of the TVM model. In future studies, researchers must validate the relevant indicators based on specific research objectives to ensure an optimal balance between research efficacy and cost-effectiveness.

## Conclusion

This study successfully modified the pre-existing rat model of POP and effectively mimicked human TVM surgery using this model. The TVM surgery not only corrected pelvic organ prolapse in rats but also offered valuable insights for future investigations into the mechanisms of pelvic mesh exposure in rats.

## Data Availability

The raw data supporting the conclusions of this article will be made available by the authors, without undue reservation.
